# Functional Analysis of Rice Long-Chain Acyl-CoA Synthetase 9 (*OsLACS9*) in the Chloroplast Envelope Membrane

**DOI:** 10.3390/ijms21062223

**Published:** 2020-03-23

**Authors:** Aya Kitajima-Koga, Marouane Baslam, Yuuki Hamada, Namiko Ito, Tomoko Taniuchi, Takeshi Takamatsu, Kazusato Oikawa, Kentaro Kaneko, Toshiaki Mitsui

**Affiliations:** 1 Laboratory of Biochemistry, Faculty of Agriculture, Niigata University, Niigata 950-2181, Japan; 2 Department of Life and Food Sciences, Graduate School of Science and Technology, Niigata University, Niigata 950-2181, Japan

**Keywords:** α-amylase, carbon metabolism, LACS9, Golgi-to-plastid trafficking, growth, *Oryza sativa*, secretory pathway, starch, subcellular localization

## Abstract

The long-chain acyl-CoA synthetases (LACSs) are involved in lipid synthesis, fatty acid catabolism, and the transport of fatty acids between subcellular compartments. These enzymes catalyze the critical reaction of fatty acyl chains to fatty acyl-CoAs for the triacylglycerol biosynthesis used as carbon and energy reserves. In *Arabidopsis*, LACSs are encoded by a family of nine genes, with LACS9 being the only member located in the chloroplast envelope membrane. However, the comprehensive role of LACS9 and its contribution to plant metabolism have not been explored thoroughly. In this study, we report on the identification and characterization of LACS9 mutants in rice plants. Our results indicate that the loss-of-function mutations in *OsLACS9* affect the architecture of internodes resulting in dwarf plants with large starch granules in the chloroplast, showing the suppression of starch degradation. Moreover, the plastid localization of α-amylase I-1 (AmyI-1)—a key enzyme involved in starch breakdown in plastids—was suppressed in the *lacs9* mutant line. Immunological and confocal laser scanning microscopy analyses showed that OsLACS9-GFP is located in the chloroplast envelope in green tissue. Microscopic analysis showed that OsLACS9s interact with each other in the plastid envelope membrane. Furthermore, OsLACS9 is also one of the proteins transported to plastids without a transit peptide or involvement of the Toc/Tic complex system. To identify the plastid-targeting signal of OsLACS9, the transient expression and localization of a series of N-terminal truncated OsLACS9-green fluorescent protein (GFP) fusion proteins were examined. Truncation analyses identified the N-terminal 30 amino acid residues to be required for OsLACS9 plastid localization. Overall, the data in this study provide an advanced understanding of the function of OsLACS9 and its role in starch degradation and plant growth.

## 1. Introduction

Plants assimilate carbon dioxide by daytime photosynthesis for the biogenesis of essential structures such as proteins, membranes, and structural carbohydrates. A portion of the photosynthetically fixed carbon in leaves is retained within the chloroplasts during the day to synthesize starch, which is then remobilized during the subsequent night to support nonphotosynthetic metabolism and growth by the continued allocation of carbon to the rest of the plant. Starch is the main storage carbohydrate in vascular plants. In the absence of photoassimilation, the transitory starch stored in the source organs is degraded to replenish cellular sugars in order to avoid carbon starvation. The starch accumulation in illuminated leaves is the result of the balance between starch synthesis and degradation, and the efficiency with which starch breakdown products are recycled back to starch. Various mechanisms have been proposed to describe starch synthesis and degradation in leaves [1,2]. Examples include simultaneous synthesis and degradation of starch in leaves in the light [3,4,5,6]. The α-amylase isoform I-1 (AmyI-1) is a key enzyme in starch metabolism and it has been reported that AmyI-1 is involved in starch degradation in plastids [7,8]. Yamaguchi et al. [9] showed a correlation between α-amylase and starch content in the leaf sheath after heading with the activity being consistent with the degree of starch degradation. AmyI-1 is also targeted to chloroplasts and plastids [7,8]. AmyI-1 is a well-characterized secretory glycoprotein bearing an *N*-linked oligosaccharide side chain and is delivered from the Golgi apparatus through the secretory pathway to the plastids in rice cells. The existence of a noncanonical protein import route into the plastids through the secretory pathway has been recently discovered [7,10,11,12]. Several glycoproteins are transported to plastids through the secretory pathway in monocot and dicot species [8,11,13,14,15,16]. However, the key role players participating in the transport of AmyI-1 and the mechanism of the membrane trafficking of proteins from the Golgi apparatus to plastids still remain to be elucidated. 

Starch granules are densely packed with semi-crystalline lamellar structures consisting of mainly linear amylose and highly branched amylopectin. In addition to the major polysaccharide components, starch granules also contain very small amounts of proteins, lipids, and phosphorus. Lipids are present in cereal starches in the form of free fatty acids and lysophospholipids [17,18]. A close relationship between the accumulation of starch granule lipids and amylose has been established and a part of these lipids is complexed with amylose helices [19]. In higher plants, de novo fatty acid synthesis (FAS), which plays essential roles in normal plant growth and development, occurs almost exclusively in the plastid. 

The fatty acids released are required to be exported from the plastids by crossing both the inner and outer envelopes of the plastid to be used for phosphatidylcholine and triacylglycerol (TAG) biosynthesis in the endoplasmic reticulum (ER). Recently, Li et al. [20] reported that *fatty acid export* (*FAX*) *1* mediates the transfer of free fatty acids across the inner envelope. The major exported products of chloroplast FAS, e.g., 18:1 and 16:0 FA, are transferred to the outer plastid envelope membranes where they are activated to acyl-CoAs by a long-chain acyl-CoA synthetase (LACSs) in the outer plastid envelope [21]. The resulting acyl-CoA is exported to the ER through the cytosol, with this transfer probably being facilitated by acyl-CoA binding proteins [22]. Alternatively, the acyl-CoA might be incorporated into phosphatidylcholine through lysophosphatidylcholine acyltransferase at the plastid envelope and then exported to the ER [23]. LACSs are AMP-binding proteins that form part of the carboxyl-CoA ligase superfamily, collectively known as acyl-activating enzymes [24]. LACSs catalyze the formation of acyl-CoA by a two-step mechanism involving in the first reaction the conversion of free fatty acid and ATP to an enzyme-bound acyl-AMP intermediate. As a second reaction, the thioester bond formation with CoA generates AMP and the acyl-CoA final product [25]. Plants contain several LACS isoforms that participate in different anabolic and catabolic processes, with their role in fatty acid transport being the most well-characterized. Various genes encoding LACS have been characterized in *Arabidopsis* [21,26,27], pea (*Pisum sativum*) [28], and oilseeds plants such as rapeseed (*Brassica napus*) [29] and sunflower (*Helianthus annuus*) [30]. For instance, nine LACS genes (*AtLACS1*–*AtLACS9*) have been described in *Arabidopsis* and their role in plant metabolism has been studied [21,26]. The encoded proteins from LACSs have enzymatic activity when expressed in *E. coli* [31]. LACSs are distributed distinctly and as demonstrated by the protein AtLACS9, these enzymes are located, among the nine family genes, on the cytosolic face of the plastid outer envelope [26,27,28,29,32]. LACSs are particularly challenging and interesting as targets for molecular analysis owing to their fundamental role in providing activated acyl groups as substrates in various fatty acid metabolic pathways [26]. Although the substrate specificity and tissue-specific expression have been evaluated for all members of the family, the biological role of individual members has been established in only a few cases [32]. For instance, the *AtLACS2* gene is required for cutin biosynthesis [21] and the *AtLACS6* and *AtLACS7* genes are involved in the breakdown of storage lipid in cells [31]. However, although the complex and highly regulated lipid metabolism plays a very important role in the normal plant growth and development, little is known about LACS9 enzymes in plants. 

Here, we provide, to our knowledge, the first insights into LACS9 function in rice. Thus, we report the characterization of the *OsLACS9* gene and we provide insight into plant AmyI-1 and LACS9 function in the network that coordinate to starch metabolism and growth. We first investigated the visual phenotypes, followed by the subcellular localization, organelle-targeting signals, and function of OsLACS9 in starch metabolism. Our results indicate that OsLACS9 has localization signals for the plastid and membrane fraction. Further, OsLACS9 directly interact with each other through the plastid membrane and via the possible association, or not, with the secretory glycoprotein AmyI-1 in the interior of plastids. Our data seem to suggest that OsLACS9 is involved in membrane trafficking through the secretory pathway to plastids in higher plant cells.

## 2. Results and Discussion

### 2.1. Loss-of-Function of OsLACS9 Affects Plant Growth

**Figure 1:**
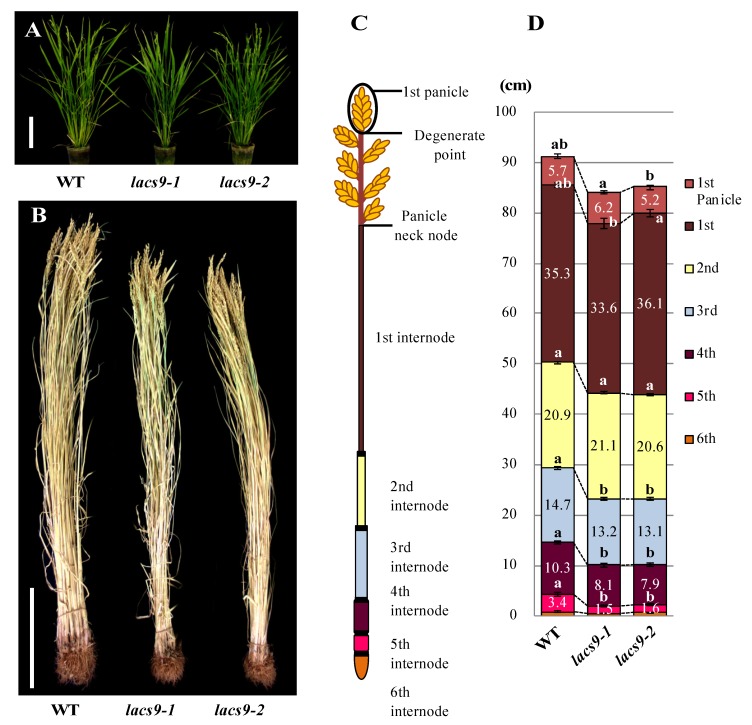
Phenotype of the wild type (WT) and *lacs9* mutant lines (*lacs9-1* and *lacs9-2*) of rice. Panel (**A**) nine-week-old WT and *lacs9* mutant lines, (**B**) growth performance of the WT and *lacs9* mutant lines. Bars = 20 cm. (**C**) Schematic representation of measurable internode pattern in rice until the panicle neck node and (**D**) internode length of WT and both *lacs9* mutants (*n* = 60). Values represent the mean ± standard deviation (SD). Within WT and each mutant lines with the same letter do not differ significantly (*P* ≤ 0.05).

The homozygous *OsLACS9* knockout mutant lines *lacs9-1* (NE3550) and *lacs9-2* (NF6026) were both characterized by reduced biomass during the developing stage at nine weeks (Figure 1A). The *Oryza sativa lacs9* mutant plants developed slower compared to the wild type (WT), were significantly reduced in size, and had shorter stems. Detailed analysis of different organs revealed that the decrease in biomass of the rice *lacs9-1* and *lacs9-2* lines was detectable throughout the entire plant body, including roots, leaves, and stem tissues (Figure 1B). To further analyze plant development, we examined the morphology of the 1st to 5th internodes from mutants (Figure 1C,D) at the mature stage. As shown in Figure 1D, the mature (dry to harvest) rice plants from the *lacs9-1* and *lacs9-2* lines showed shorter internode lengths compared to WT, with the difference being greatest at the 5th internode. Since the same phenotype was observed in both the independent T-DNA insertion rice lines *lacs9-1* and *lacs9-2* (Figure 1 A–D), we conclude that the obvious morphological defects and striking phenotype were caused by the loss of *OsLACS9* function. Since *LACS9* in rice belongs to a family of nine proteins (Figure S1), the plastid-predicted *OsLACS1-8* whose expression is regulated throughout plant development, most likely cannot compensate for the loss of *OsLACS9* function in all tissues and organs, thus leading to the restoration of the rather mild overall phenotype of the *lacs9* knockouts. The investigation of plant *LACSs* has lagged behind that of mammals, yeast, and bacteria. In *Arabidopsis*, nine LACS genes (*AtLACSs*) encoding enzymes with distinct tissue distributions, subcellular locations, and biological functions have been identified [26]. The *Arabidopsis lacs9-1* mutant was indistinguishable from the WT in size and appearance and produced normal-looking seed with WT amounts of fatty acids [21]. Data have suggested that, in *Arabidopsis*, one or more additional LACS isoforms must be active in the plastid. Mutation in the LACS1 gene of *Arabidopsis* revealed a role for LACS1 in the biosynthesis of cuticular wax components [33]. Previous reports demonstrated that double mutation in *lacs1 lacs2*, which were shown to reside in the ER, appear to have an overlapping function in the biosynthesis of wax and long-chain (C_16_) fatty acids for cutin synthesis [34,35]. Double mutation in *lacs1 lacs2* reduced the amount of wax and cutin and thus plants displayed abnormal phenotypes not found in either of the parental mutants. Indeed, *lacs1 lacs2* double-mutant plants displayed pleiotropic phenotypes including organ fusion, abnormal flower development, reduced seed set, and susceptibility to drought stress [33]. AtLACS4, also located in the ER, was shown to be possibly involved in the synthesis of surface lipids [32]. Jessen et al. [32] found AtLACS1 and AtLACS4 to have a crucial role in the formation of pollen coat lipids in *Arabidopsis*. In contrast, AtLACS6 and AtLACS7, which were found to be located in the peroxisome, were suggested to be required for the activation of fatty acids for β-oxidation and successful seedling development [31,36]. The single-mutant lines, *lacs6* and *lacs7*, were indistinguishable from the wild type in germination, growth, and reproductive development [36]. While the *lacs6 lacs7* double-mutant grown on media without sucrose showed white and unexpanded cotyledons, the growth and development of these seedlings on sucrose closely matched those of wild type controls (with or without sucrose) [37]. 

It was shown, in *Arabidopsis*, that AtLACS9 is the only LACS to reside in the plastid, the site of de novo fatty acid synthesis, and thus was considered as the major candidate for activating and exporting plastidial-derived fatty acids for TAG formation [38]. Although the plastidial LACS activity in the *lacs9* null mutant of *Arabidopsis* decreased by 90%, the mutant plant did not display any detectable phenotype, suggesting that (1) additional LACS isoforms are involved in exporting plastidial fatty acids for TAG biosynthesis (2) another parallel pathway or pathways or mechanisms might be required for successful growth beside the flux of fatty acids in *lacs* mutant plants. Jessen et al. (2015) reported that AtLACS9 might contribute to lipid trafficking from the ER back to the plastid rather than fatty acid export outside the plastid [32].

### 2.2. Chloroplasts of Mutants Lacking OsLACS9 Contain Large Starch Granules

**Figure 2:**
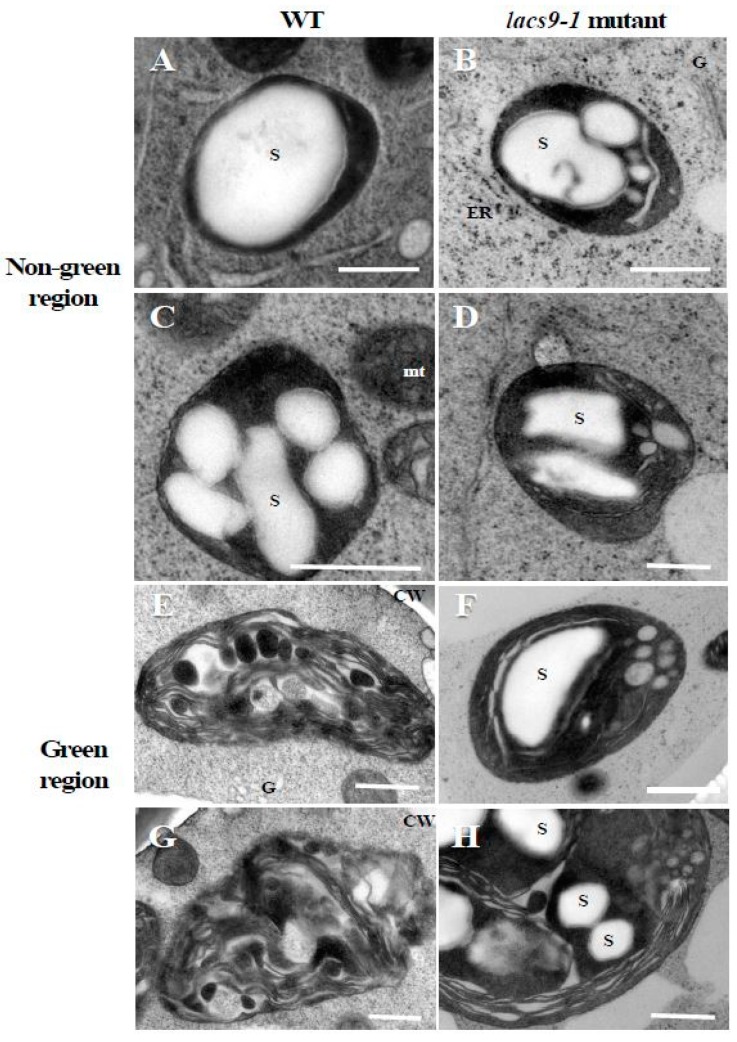
Transmission electron microscope analysis in WT and *lacs9-1* mutant cells of rice. Panels (**A**,**C**,**E**,**G**) represent WT, and (**B**,**D**,**F**,**H**) represent *lacs9* mutant cultured rice cells. (**A**–**D**) Nongreen region, (**E**–**H**) green region. S: Starch granule; CW: Cell wall; ER: Endoplasmic reticulum; G: Golgi apparatus; mt: Mitochondria. Bars = 0.5 µm.

To elucidate the underlying molecular and cell biology aspects of the observed visual phenotypes, starch structure was analyzed. As shown in Figure 2A–D, transmission electron microscope analysis in WT and *oslacs9* knockout cells showed that their plastids were quite similar, with both containing large starch granules in nongreen (colourless) cultured rice cells. However, in differentiated green cells (Figure 2E–H), differences from WT cells were observed in mutant lines. While in WT the thylakoid membrane was developed and starch granules were smaller as compared to the nongreen region (Figure 2E,G), the *lacs9* mutant still showed the existence of large starch granules (Figure 2F,H). Further, the *lacs9* plastids in greening cells were similar to those in colourless region cells as compared to the green region plastids of the WT.

We have previously reported the involvement of AmyI-1, a well-characterized secretory glycoprotein, in starch degradation in the plastids of rice cells [8], and its role as a key enzyme in starch degradation in germinating seeds. Kitajima et al. [7] demonstrated in rice and onion that AmyI-1 is targeted to the plastid from the Golgi apparatus through the secretory pathway. In this study, the TEM observation results suggested that loss-of-function of *OsLACS9* affected starch metabolism in chloroplasts. Thus, we considered the possibility that OsLACS9 might affect starch metabolism-related proteins such as AmyI-1. Earlier studies showed that the two glycoproteins carbonic anhydrase [13] and rice nucleotide pyrophosphatase/phosphodiesterase [15] were targeted to plastids in a Brefeldin A-sensitive manner [38]. The findings from these studies suggested that membrane trafficking is necessary for plastid targeting of these glycoproteins. Three-dimensional time-lapse confocal imaging demonstrated that the Golgi membrane vesicles appear to be taken up and spread inside the plastid [7]. Moreover, electron microscopy observations of quick-frozen cells showed the moment membrane vesicles entered the plastids as the vesicles were passed through the plastid envelope membranes. These observations indicate that a novel vesicle-trapping mechanism operates in AmyI-1 importation into plastids [39].

### 2.3. Prevention of Chloroplast Localization of AmyI-1 in lacs9 Mutant Cells

**Figure 3:**
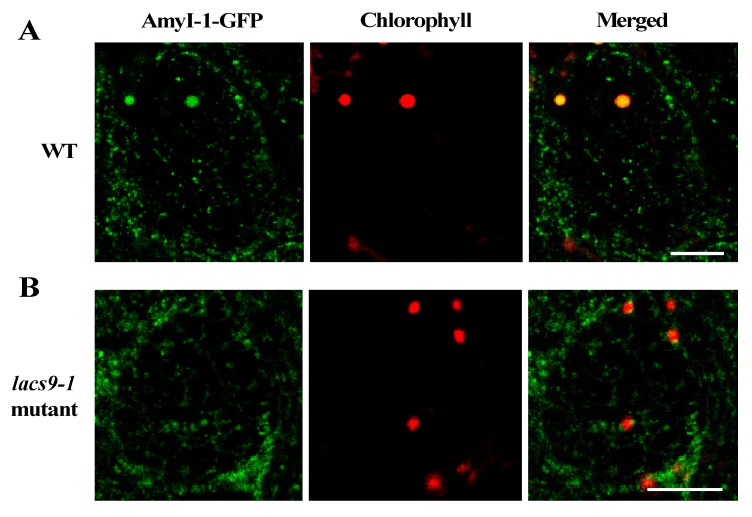
Prevention of α-amylase isoform I-1 (AmyI-1) chloroplast localization in *lacs9-1* mutant cells. Confocal laser scanning-microscopic images of chloroplasts in (**A**) WT and (**B**) *lacs9-1* rice cells with expression of AmyI-1-green fluorescent protein (GFP). Red and green colors show the fluorescence signals of chlorophyll and GFP, respectively. Bars = 5 μm.

To investigate whether the loss-of-function of *OsLACS9* affects AmyI-1 targeting to the plastid and the overlapping functions, the subcellular localization of AmyI-1 and OsLACS9 proteins was investigated in greening cultured rice cells (Figure 3). First, we stably transformed rice cells with AmyI-1-GFP in WT and *lacs9* mutant cells. As shown in Figure 3A, AmyI-1-GFP was located in the chloroplasts as visualized by chlorophyll autofluorescence in WT cells. In contrast, virtually no AmyI-1 was found in the plastids in *lacs9* mutant cells (Figure 3B). The fluorescent AmyI-1-GFP was located in the cytoplasm. This result confirmed that the localization of AmyI-1-GFP in plastids was suppressed in *lacs9* cells. This could be explained, at least in part, by the existence of the large starch granules in *lacs9* chloroplasts.

### 2.4. OsLACS9 is Localized to Both Plastids and The Membrane Fraction

**Figure 4:**
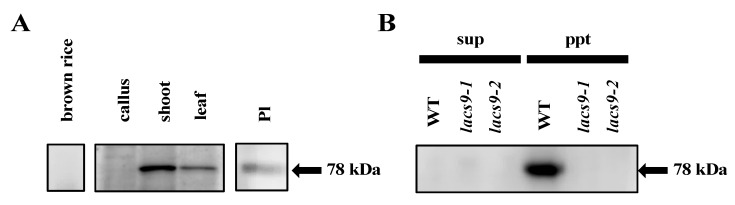
Long-chain acyl-CoA synthetase 9 (OsLACS9) localized to chloroplasts and membrane fraction. Immunoblot detection of OsLACS9. (**A**) Proteins (20 µg) extracted from brown rice, callus, shoot, leaf, and intact plastid (Pl) isolated from shoots of WT line. (**B**) The soluble (sup) and membrane pellet (ppt) fractions of WT and *lacs9* mutant lines shoot subjected to sodium dodecyl sulfate-polyacrylamide gel electrophoresis (SDS-PAGE) using anti-OsLACS9 antibodies. The position of protein size markers (in kDa) is indicated at the right (black arrow).

To assess the subcellular localization and function of OsLACS9, we immunoblotted seeds, calli, shoots, and mature leaves with antibodies specific to the OsLACS9 peptides. Our result, first, confirmed the suppression of *OsLACS9* in the *lacs9* mutant lines. In the WT plants, the OsLACS9 protein had been identified in the membrane fraction. However, it was not detected in the soluble and membrane fraction in either the *lacs9-1* or *lacs9-2* mutant lines (Figure 4B). Second, the data showed that the protein bands recognized with anti-OsLACS9 antibodies accumulated much more in the green tissues, shoots, and leaves (Figure 4A). Further, cell fractionation analysis of leaf extract in the membrane and soluble fractions indicated that OsLACS9 was detected only in the membrane fraction of WT plants (Figure 4B). The results suggest that OsLACS9 is a membrane protein. Indeed, this enzyme is membrane-bound and it contains two transmembrane domains in its sequence (Figure S2). We also examined the localization of OsLACS9 in isolated chloroplasts. The data from the immunoblotting analysis using the anti-OsLACS9 antibodies confirmed the localization of LACS9 in the plastids (Figure 4C). Localization of LACS9 in the outer plastid envelope has been established in *Arabidopsis* [21]. Altogether, our results confirmed that OsLACS9 is a chloroplast membrane protein.

### 2.5. OsLACS9-GFP is Located in The Envelope Membrane of Rice Chloroplast 

**Figure 5:**
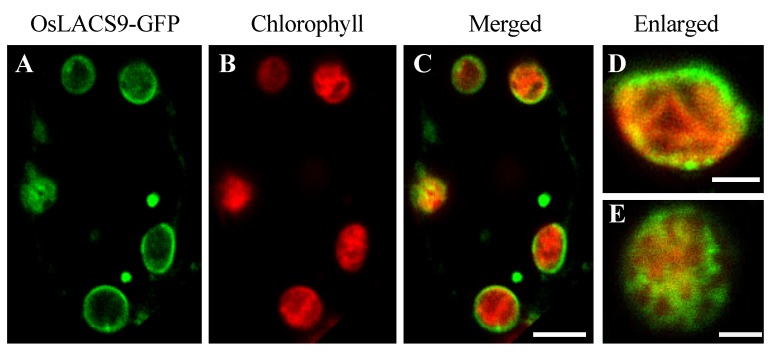
Subcellular localization of LACS9 in leaf mesophyll cells of rice stably expressing OsLACS9-GFP. Confocal fluorescence microscopy images show (**A**) the signal of OsLACS9-GFP, (**B**) the chlorophyll autofluorescence, (**C**) the merged images, and (**D**,**E**) enlarged images of the chloroplast. Bars = 5 µm.

In the present study, we sought to further elucidate the subcellular localization. To visualize the localization of OsLACS9 proteins within the cell, fluorescent fusion proteins were constructed. The rice cells stably expressing OsLACS9-GFP were obtained by transformation with pZH2B-35S-OsLACS9-GFP containing *CaMV35S* driven *OsLACS9* fused *GFP* and *mHPT* genes. Using the transgenic cultured cells thus obtained, the subcellular localization of OsLACS9-GFP was examined with confocal laser-scanning microscopy (CLMS). As shown in Figure 5, the expression levels of LACS9 in rice were observed on the chloroplast surface, resulting in the ring-like appearance of fluorescence signals (green) surrounding the chloroplasts (displayed in red). Additionally, the presence of a fluorescence signal was located in the vesicle-like structures (Figure 5 A–C). These observations indicate that the OsLACS9-GFP localization on the plastid envelope membrane of leaf mesophyll cells was consistent with the immunoblot analysis shown in Figure 4.

Enlarged images of the chloroplasts (Figure 5D,E) showed that the OsLACS9 protein was organized in a mesh-like structure on the chloroplast surface. These results indicate that the OsLACS9 proteins have significant functional roles in the chloroplasts and do not exist evenly. In *Arabidopsis*, previous studies suggested that LACS9 resides in the plastid envelope, while LACS1, 4, and 8 were located in the ER, and LACS6 and 7 in the peroxisome [21,26,35]. Comparison of the LACS9 families showed that LACS8 had a high sequence similarity to LACS9 and based on in vitro assays, LACS8 was predicted to be the most likely candidate for exercising an active role in the plastid [21,26]. To test this hypothesis, we considered that its function was to divide the organelles in which the LACS family members reside. Recently, research into contact sites or “communication zones” that enable the exchange of molecules among different organelles, e.g., ER-Golgi apparatus, ER-mitochondria have been gaining more attention. It should be noted that the distribution of membrane components and transport devices in organelles is biased, suggesting that functions are shared in each area. In Figure 5D,E, the OsLACS9-GFP was not located uniformly throughout the plastid envelope membrane, and mesh-like structures were observed. These findings suggest the possibility that OsLACS9 has a function at the contact site between organelles, and this is consistent with a report in animal cells suggesting that the OsLACS9 homolog acyl-CoA synthetase ligases (ACSL4) are present in the mitochondria-associated membrane (MAM) [40]. 

### 2.6. OsLACS9 Interacts with Each Other on Plastid Envelope Membrane

**Figure 6:**
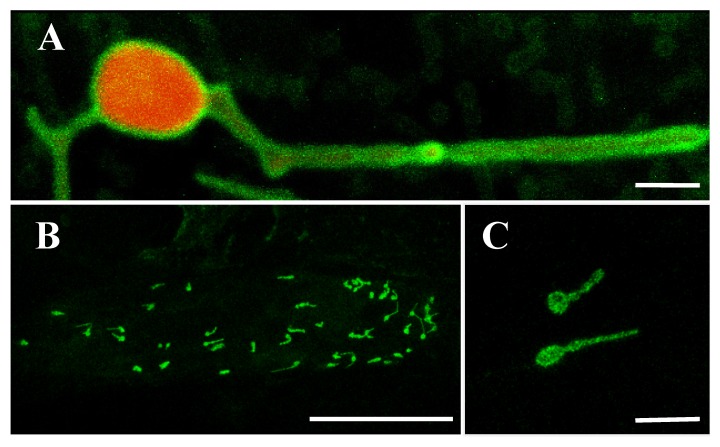
BiFC analysis of OsLACS9-nYFP and OsLACS9-cYFP. Images show onion epidermal cells expressing: (**A**) Transient expression of OsLACS9-GFP (green) and WxTP-DsRed (plastid stroma marker, red). Bar = 2 µm. (**B**,**C**) OsLACS9-nYFP and OsLACS9-cYFP. The green color represents nYFP/cYFP complex. Bars in (**B**,**C**) represent 100 and 10 µm, respectively.

The *OsLACS9* homolog, ACSL4, has been reported to be present in MAM; the contact site between the ER and the mitochondria. To elucidate the possibility that OsLACS9 is involved in the transport of secretory proteins such as AmyI-1 to the plastid, we carried out a bimolecular fluorescence complementation (BiFC) assay to look for a possible interaction between AmyI-1 and OsLACS9. WxTP-DsRed was co-expressed as a plastid stroma marker owing to the absence of chlorophyll autofluorescence in onion epidermal cells. Our results showed that the green fluorescence of OsLACS9-GFP was located on the plastid surface and it was diffused among the stromal component labeled with the red fluorescent (Figure 6A). These data corroborate the finding in rice cells which showed that OsLACS9-GFP was located in the plastid envelope membrane. Subsequently, the BiFC assay was performed using multiple combinations: OsLACS9-N-terminal portion of the yellow fluorescent protein (nYFP) and AmyI-1-C-terminal portion of the yellow fluorescent protein (cYFP), OsLACS9-cYFP and AmyI-1-nYFP, or OsLACS9-nYFP and OsLACS9-cYFP. The reconstituted YFP signals were detected in the chloroplast envelope membrane when OsLACS9-nYFP and OsLACS9-cYFP were co-expressed (Figure 6B,C), clearly indicating that OsLACS9s interact with each other in the plastid envelope membrane. In contrast, the YFP signal was scarcely detectable in both combinations of OsLACS9 and AmyI-1, suggesting that direct interaction between OsLACS9 and AmyI-1 is unlikely. Our results suggest that OsLACS9 might be involved in the plastid targeting mechanism of AmyI-1 although we consider that other components, which still remain to be elucidated, are needed for the plastid targeting of AmyI-1 given that OsLACS9 did not directly interact with AmyI-1. 

### 2.7. N-terminal Sequence of OsLACS9 is Necessary for Targeting to Chloroplasts in Onion Cells

**Figure 7:**
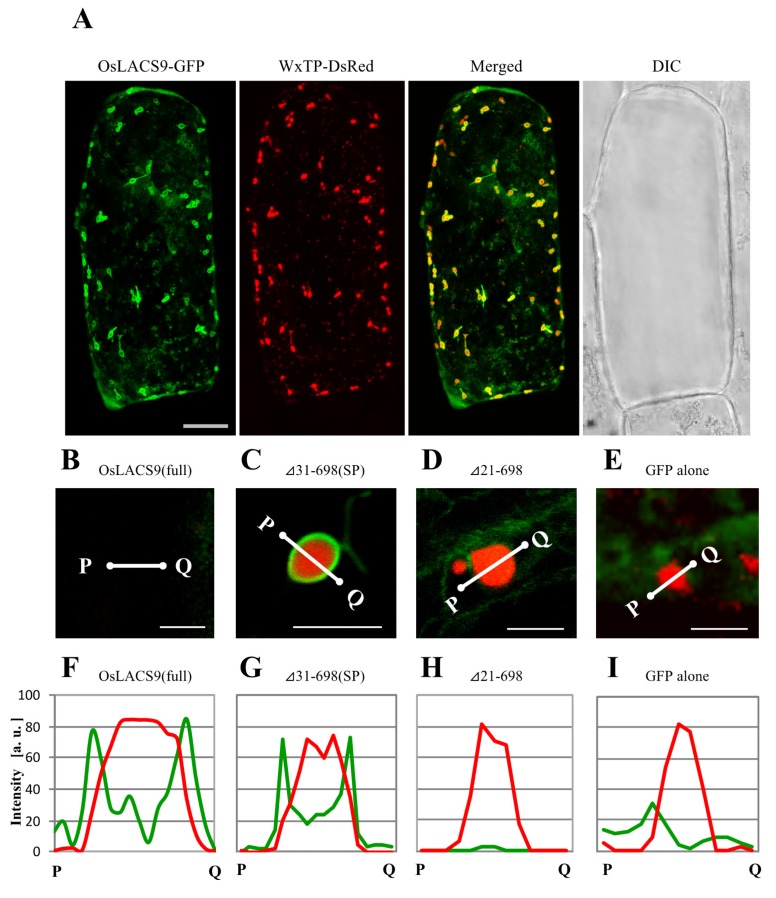
Confocal imaging of onion epidermal cells transiently co-expressing truncated OsLACS9-GFP and WxTP-*Discosoma* sp. red fluorescent protein (DsRed). (**A**) OsLACS9-GFP and transit peptide of rice granule-bound starch synthase 1 (WxTP)-DsRed were co-expressed in onion epidermal cells by using the particle-delivery system and subjected to confocal laser scanning microscopy. Merged: OsLACS9-GFP+WxTP-DsRed; DIC: Differential interference contrast image. Bars = 25 μm. Enlarged images of plastids co-expressing full OsLACS9-GFP (**B**); truncated LACS_31-698_. SP: Signal peptide (**C**); LACS_22-698_ (**D**); or GFP alone (**E**). Bars = 5 μm. (**F**–**I**) Distribution profiles of OsLACS9-GFP fluorescence in plastid visualized by the plastid marker WxTP-DsRed. The intensity of GFP (green lines) and DsRed (red lines) fluorescence was analyzed using the ImageJ software program. a. u.: Arbitrary unit.

To assess the organelle-targeting signals of OsLACS, we examined the transient expression and localization of a series of carboxy-terminal truncated OsLACS9 labeled with GFP in bombarded onion cells using CLSM. In particular, we examined the full-length and the truncated C-terminal fragment comprising Δ41-698, Δ31-698, and Δ21-698 labeled with GFP (Figure 7A). Simultaneous expression of OsLACS9-GFP and WxTP-DsRed were found surrounding the plastid through its predicted C-terminal (Figure 7A). Enlarged images of the plastid (Figure 7B) expressing the full-sequence of OsLACS9-GFP clearly show its localization on the surface of the plastid in onion epidermal cells. Similar data were observed in cultured rice cells (Figure 5). Within the C-terminal fragment truncations, the subcellular distribution of Δ31-698 (Figure 7C) or longer sequences (Figure S3) were observed throughout the plastid, with a similar distribution to that of the full OsLACS9-GFP (Figure 6A and Figure 7B). In contrast, the shorter Δ21-698 (Figure 7D) and ΔSP (Figure S3G) sequences of OsLACS9 lost the ability to target plastids. We further checked the distribution profiles of OsLACS9-GFP fluorescence intensities in the regions close to the plastids (Figure 7F–I). In this way, as shown in Figure 7F,G, the fluorescence intensities of the two green peaks were observed at both sides of the red peak, suggesting that the OsLACS9-GFP containing a 30 amino acid sequence from the N-terminus is located around the plastids. This region, 30 amino acid residues at the N-terminal of OsLACS9, functions as the predicted ER retention signal peptide necessary for targeting the OsLACS9 to the plastids. Unlike the majority of chloroplast proteins synthesized in the cytosol as precursors with an N-terminal transit peptide (TP) and are post-translationally imported into organelles via the translocon at the outer/inner chloroplast envelope (Toc/Tic) complex, OsLACS9 contains a predicted ER signal peptide (SP) sequence at the N-terminal rather than TP. Taking these data together, we postulate that OsLACS9 is one of the proteins imported into the plastid without TP and is not mediated by the Toc/Tic complex. 

## 3. Materials and Methods 

### 3.1. Plant Materials and Growth Conditions 

The rice used in this study was *Oryza sativa* L. cv. Nipponbare (WT). The *Tos17*-inserts lines of *OsLACS9* (*lacs9-1*; NE3550 and *lacs9-2*; NF6026) (Figure S4) were obtained from the National Institute of Agrobiological Sciences (NIAS, Tsukuba, Japan). The genotyping primers used for each line are summarized in Supplementary Table S1. WT and *lacs9* mutant plants were grown and harvested in the Niigata University paddy field (Niigata, Japan). We grew WT and *lacs9* mutant plants in the paddy fields of the Crop Research Center, Niigata University, Japan (37° 51’ 20.75” N 138° 57’ 37.9” E), during May–September. Thirty-day-old seedlings were transplanted at a spacing of 20 x 15 cm and grown to collect the seeds for the subsequent analyses.

The WT and *lacs9* mutant seeds were grown in a commercial soil (Kumiai Gousei Baido 3, JA, Tokyo, Japan) in plastic pots and incubated in the growth chamber (CFH-415, Tomy Seiko, Tokyo, Japan) at 26 °C (12 h day)/23 °C (12 h night) cycles with 20,000 lux of fluorescent lighting, as described previously [14], to evaluate the phenotype trait. The experiment was laid out in a randomized complete block design with six replications. Four uniform looking plants from each rice line were selected to determine the phenotype.

Seeds and plant samples were stored at 4 °C before analysis.

### 3.2. Plasmid Construction

All plasmids and primer sequences for the PCR amplifications used in this study and references describing how they were constructed are listed in Table S2. The constructions of pAmyI-1, pGFP, and pAmyI-1-GFP have been described previously [7,8]. For DsRed2 expression in onion epidermal cells, the BamHI-SacI PCR-amplified fragment from pDsRed2 (Takara Bio, Ohtsu, Japan) was cloned into the same sites of pGFP to produce pDsRed. To create pWxTP-DsRed, we PCR-amplified the first 1-111 amino acid residues, including the transit peptide sequence, from the rice waxy gene (PWCW) [7] with two flanking primers, and then digested the PCR product with BamHI. The BamHI-digested fragment was inserted into the same site as pDsRed.

For OsLACS9-GFP expression in onion and rice cells, the BamHI-KpnI PCR amplified fragment of OsLACS9 (AK065718—Os12g0168700) was cloned into pGFP. For BiFC analysis, we constructed pOsLACS9-n/cYFP and pAmyI-1-n/cYFP. PCR amplified a fragment of OsLACS9 and AmyI-1 was cloned into the pGWn/cYFP vector (AB626693, AB626695) using the Gateway system (Invitrogen, Carlsbad, CA, USA).

### 3.3. Binary Vector Constructions and Plant Transformations 

The pZH2B-35S-AmyI-1-GFP-NOS was constructed as described previously [8]. To make the transgenic line expressing OsLACS9-GFP, the BamHI-KpnI PCR-amplified fragment of OsLACS9 was cloned into pZH2B-35S-GFP-NOS.

Agrobacterium-mediated transformation and regeneration of rice plants were performed according to the methods described by Hiei et al. [41] and Fukuoka et al. [42]. 

### 3.4. Subcellular Localization

Subcellular localization was investigated by generating stable transgenic rice lines. To establish stably transformed rice lines expressing LACS-GFP fusion proteins, pZH2B-35S-LACS9-GFP was transformed into *A. tumefaciens* EHA105 and used for the transformation of rice WT plants. pZH2B-35S-AmyI-1-GFP was transformed into *A. tumefaciens* EHA105 and used for the transformation of rice *lacs9* mutant plants. The stable transformant cells were sectioned with a vibratome to a thickness of 25 μm, and immediately observed by means of confocal laser scanning microscopy.

### 3.5. Transient Expression Analysis

Transient expression assays used epidermal onion cells (*Allium cepa*) and rice seedlings on 1% agar. Particle bombardment was carried out with a helium-driven particle accelerator (PDS-1000/He; Bio-Rad) as described previously [7]. Briefly, 3 µg of plasmid DNA in 10 µI of distilled water were mixed with 10 µL of a 60 mg/mL gold particle (diameter 1.0 urn) solution, 10 µL of 2.5 mM CaCl_2_, and 4 µL of 0.1 M spermidine. The resulting mixture was incubated for 30 min at room temperature. Gold particles coated with plasmid DNA were rinsed with cold ethanol and then gently suspended in 10 µL of ethanol. The gold particles were bombarded twice in onion cells using the particle delivery system with 1100 psi rupture discs. The bombarded cells were cultured on 1% agar with a Murashige and Skoog medium at 23 °C (onion) or 28 °C (rice) in darkness, and then observed by using a confocal laser scanning microscope.

### 3.6. Microscopic Studies 

#### 3.6.1. Confocal Laser Scanning Microscope (CLSM)

Confocal laser scanning microscopy was used to analyze transient expression in onions and stable transgenic rice plants using an SP8 confocal laser scanning microscope (Leica Microsystems, Wetzlar, Germany). The Ar and green He/Ne lasers were used to excite GFP at 488 nm, DsRed at 543 nm, and 561 nm for chlorophyll autofluorescence.

#### 3.6.2. Transmission Electron Microscope (TEM) Observation

To investigate the changes in starch granules in the WT and *lacs9* mutant cells, the leaf tissues were prepared for electron microscopy. The cultured cells used for microscopic analysis were obtained from rice *calli* derived from the embryo portions of previously sterilized seeds and cultured as specified in Mitsui et al. [43] and Kaneko et al. [44]. Callus cells were grown in a Sakaguchi flask in a Murashige and Skoog (MS) medium containing 3% (w/v) sucrose, 2 mg/l 2,4-D, and 5 mg/l thiamine-HCl, placed on a reciprocal shaker operated at 110 strokes min^−1^ with a 70 mm amplitude, at 28 °C in darkness. The established suspension-cultured cells were subcultured at seven-day intervals. All these procedures were performed under aseptic conditions. The TEM analysis of the WT and transgenic rice cells expressing ST-GFP without expression of *OsLACS9* (*lacs9-1* line) was carried out as described previously [45]. WT and *lacs9* mutant cells were immediately placed on a flat specimen carrier and frozen in a high-pressure freezer (EM-PACT; Leica Microsystems). The frozen samples were fixed in anhydrous acetone containing 2% osmic acid (OsO4) for 3–4 days at −80 °C for morphological observation. Tubes containing the frozen samples were warmed at 3 °C/h to a temperature of −20 °C, and at 1 °C/h from −20 to 4 °C, and kept for 2 h at 4 °C using an automatic freeze substitution system (EM-AFS; Leica Microsystems). The samples were then washed with 100% acetone and embedded in epoxy resin Epon 812 (Shell Chemicals, Hague, Netherlands). Resin sections (200 nm) were cut with a diamond knife using Ultracut UCT. Ultrathin sections were stained with 2% *w/v* uranyl acetate. After staining, samples were examined with TEM (H-7650, Hitachi, Tokyo, Japan) at 80 kV. Images were acquired using a Gatan DualView camera and Digital Micrograph software or transmission electron microscopy films. 

### 3.7. Protein Extraction and Immunoblotting Analysis

Intact chloroplasts were purified from seedlings grown in a light condition using the Percoll (GE Healthcare) density-gradient centrifugation method, as described earlier [45,46] and analyzed by SDS-PAGE and immunoblotting. Proteins were extracted from brown rice seed (harvested in the field), calli (cultured in a dedifferentiation medium for four weeks), shoot (germinated at 30 °C for seven days in the dark and moved to a growth chamber (28:23 °C, 12:12 h, light:dark; 20,000 lux) for three days), and mature leaves (germinated at 30 °C for seven days in the dark and moved to a growth chamber (28:23 °C, 12:12 h, light:dark; 20,000 lux) for eight days). The samples (20 µg of protein extracts) were mixed two times with a Laemmli buffer and separated on 10% (*w/v*) SDS-PAGE. The proteins were transferred to a nitrocellulose membrane, blocked with 2% (*w/v*) dried milk in a buffer of 10 mm Tris-HCl, pH 8.0, 150 mm NaCl, and 0.25% (*v/v*) Tween 20 (TBS-T) for 1 h, and then incubated with primary antibodies at various dilutions overnight at 4 °C. After four washes in TBS-T for 10 min each, the membranes were exposed to poly-horseradish peroxidase-conjugated anti-rabbit IgG (diluted 1:5,000) (Nakarai tesque, Kyoto, Japan) for 2 h at 20 °C. After five washes with TBS-T, the signals were detected by a 3:1 mixture of SuperSignal West Pico:Femto Chemiluminescent Substrates (Pierce) (Thermo Fisher Scientific, Waltham, MA, USA). The immunoblotting analyses were carried out as described by Mitsui et al. [43]. The antibody against OsLACS9 peptides (37Glu-53Thr) was used for the immunoblot analysis. All procedures were performed at 4 °C.

### 3.8. Computer Analyses

Rice *LACS9* homologs (Table S3) were identified by a BLAST search of the RAP-DB database (https://rapdb.dna.affrc.go.jp/index.html). *Arabidopsis LACS* homologs (Table S3) were searched for in the *Arabidopsis* Information Resource (TAIR) (https://www.arabidopsis.org). In the *LACS9* family phylogenetic tree (Figure S1) comparisons of *Oryza sativa* and *Arabidopsis* were constructed by neighbor-joining methods in a CLC sequence viewer 8.0 (https://www.qiagenbioinformatics.com). The phylogenetic tree of the multiple sequence alignment of LACS proteins from different species was constructed by neighbor-joining algorithms using ClustalW in MEGA 7 using the default settings.

### 3.9. Statistical Analyses

Presented data are the means ± standard deviation. The significance of differences between WT and lacs9 mutant lines was statistically evaluated with Tukey’s HSD (Honestly Significant Difference) procedure, and differences were considered significant at *p* < 0.05 using the R software.

## 4. Conclusions

**Figure 8:**
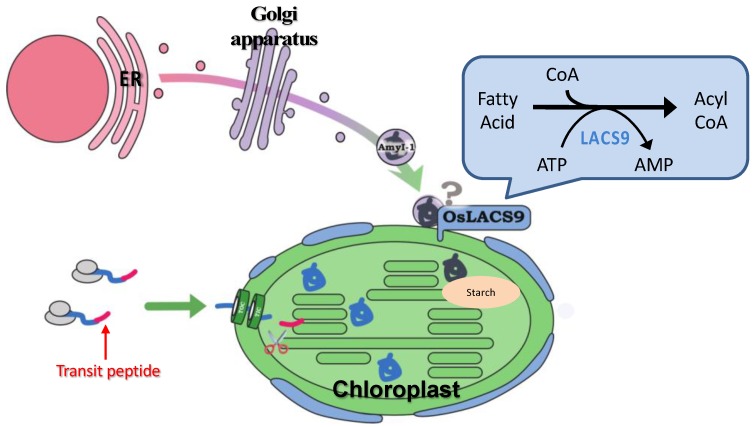
Hypothetical model of plastid targeting of glycoproteins through the secretory pathway. OsLACS9 is localized exclusively in the plastid envelope of leaf mesophyll cells and nearby vesicle-like structures. OsLACS9s interact with each other and with as yet unidentified components that might be involved in the plastid targeting mechanism of glycoproteins (i.e., AmyI-1) via the secretory pathway. According to this suggested model, OsLACS9 may permit efficient starch degradation and contribute to optimal plant growth.

A LACS-mediated plastidial lipid biosynthesis pathway has been documented, although the precise mechanism of fatty acid transport through the plastidial membrane still remain unknown. Despite ER to plastid lipid trafficking, our data point to an additional role for *O. sativa* LACS9 in starch metabolism. The results of this study provide insight into the localization of OsLACS-GFP in the plastid envelope membrane in rice and onion. The plastid localization of AmyI-1 was prevented by the loss-of-function of OsLACS9, suggesting that OsLACS9 might be involved, at least partly, in recruiting AmyI-1 to plastids through the secretory pathway, allowing efficient starch breakdown and helping to determine plant growth performance. A suggested model for involvement of OsLACS9 in plastid protein targeting through the secretory pathway is presented in Figure 8. Detailed analysis of additional possible players that function with LACS9 in AmyI-1 control mechanisms will help fine-tune current attempts at manipulating the pathway. Our findings pave the way for a better understanding of LACS protein functions in lipid and carbohydrate metabolisms as an initial step to the development of crops for the future.

## Supplementary Materials

The supplementary material for this article can be found online at https://www.mdpi.com/1422-0067/21/6/2223/s1. Table S1: Primers used for genotyping Tos17 insertion in rice lacs9 mutants. Table S2: Plasmids and primers used in this study. Table S3: Database entries of LACS from rice and Arabidopsis. Figure S1: Amino acid sequence analysis of OsLACS9 proteins. Figure S2: Structural organization of OsLACS9. Figure S3: Localization of each truncated OsLACS9-GFP and WxTP-DsRed (plastid stroma marker) fusion protein co-bombarded in onion epidermal cells. Figure S4: Genetic information on rice lacs9-1 and lacs9-2.

## References

[1] Streb S., Zeeman S.C. (2012). Starch metabolism in arabidopsis. Arab. Book.

[2] Bahaji A., Li J., Sánchez-López Á.M., Baroja-Fernández E., Muñoz F.J., Ovecka M., Almagro G., Montero M., Ezquer I., Etxeberria E. (2014). Starch biosynthesis, its regulation and biotechnological approaches to improve crop yields. Biotechnol. Adv..

[3] Baslam M., Baroja-Fernaândez E., Ricarte-Bermejo A., Sánchez-López A.M., Aranjuelo I., Bahaji A., Muñoz F.J., Almagro G., Pujol P., Galarza R. (2017). Genetic and isotope ratio mass spectrometric evidence for the occurrence of starch degradation and cycling in illuminated Arabidopsis leaves. PLoS ONE.

[4] Caspar T., Lin T.P., Kakefuda G., Benbow L., Preiss J., Somerville C. (1991). Mutants of Arabidopsis with altered regulation of starch degradation. Plant Physiol..

[5] Stitt M., Heldt H.W. (1981). Simultaneous synthesis and degradation of starch in spinach chloroplasts in the light. Biochim. Biophys. Acta Bioenerg..

[6] Szecowka M., Heise R., Tohge T., Nunes-Nesi A., Vosloh D., Huege J., Feil R., Lunn J., Nikoloski Z., Stitt M. (2013). Metabolic fluxes in an illuminated arabidopsis rosette. Plant Cell.

[7] Kitajima A., Asatsuma S., Okada H., Hamada Y., Kaneko K., Nanjo Y., Kawagoe Y., Toyooka K., Matsuoka K., Takeuchi M. (2009). The rice alpha-amylase glycoprotein is targeted from the Golgi apparatus through the secretory pathway to the plastids. Plant Cell.

[8] Asatsuma S., Sawada C., Itoh K., Okito M., Kitajima A., Mitsui T. (2005). Involvement of alpha-amylase I-1 in starch degradation in rice chloroplasts. Plant Cell Physiol..

[9] Yamaguchi N., Suzuki S., Makino A. (2013). Starch degradation by alpha-amylase in tobacco leaves during the curing process. Soil Sci. Plant Nutr..

[10] Baslam M., Oikawa K., Kitajima-koga A., Kaneko K., Mitsui T. (2016). Golgi-to-plastid traf fi cking of proteins through secretory pathway: Insights into vesicle-mediated import toward the plastids Golgi-to-plastid traf fi cking of proteins through secretory pathway: Insights into vesicle-mediated import toward the plasti. Plant Signal. Behav..

[11] Burén S. (2010). Targeting and Function of CAH1—Characterization of a Novel Protein Pathway to the Plant Cell Chloroplast.

[12] Gagat P., Bodył A., Mackiewicz P. (2013). How protein targeting to primary plastids via the endomembrane system could have evolved? A new hypothesis based on phylogenetic studies. Biol. Direct.

[13] Villarejo A., Burén S., Larsson S., Déjardin A., Monné M., Rudhe C., Karlsson J., Jansson S., Lerouge P., Rolland N. (2005). Evidence for a protein transported through the secretory pathway en route to the higher plant chloroplast. Nat. Cell Biol..

[14] Shiraya T., Mori T., Maruyama T., Sasaki M., Takamatsu T., Oikawa K., Itoh K., Kaneko K., Ichikawa H., Mitsui T. (2015). Golgi/plastid-type manganese superoxide dismutase involved in heat-stress tolerance during grain filling of rice. Plant Biotechnol. J..

[15] Nanjo Y., Oka H., Ikarashi N., Kaneko K., Kitajima A., Mitsui T., Muñoz F.J., Rodríguez-López M., Baroja-Fernández E., Pozueta-Romero J. (2006). Rice plastidial N-glycosylated nucleotide pyrophosphatase/phosphodiesterase is transported from the ER-golgi to the chloroplast through the secretory pathway. Plant Cell.

[16] Li Y.-Z., Zhao J.-Y., Wu S.-M., Fan X.-W., Luo X.-L., Chen B.-S., Anderson J.V., Ceballos H., Jansson C., Westerbergh A. (2016). Characters related to higher starch accumulation in cassava storage roots. Sci. Rep..

[17] Li H., Gidley M.J., Dhital S. (2019). High-amylose starches to bridge the “Fiber Gap”: development, structure, and nutritional functionality. Compr. Rev. Food Sci. Food Saf..

[18] Morrison W.R., Milligan T.P., Azudin M.N. (1984). A relationship between the amylose and lipid contents of starches from diploid cereals. J. Cereal Sci..

[19] Pérez S., Bertoft E. (2010). The molecular structures of starch components and their contribution to the architecture of starch granules: A comprehensive review. Starch Stärke.

[20] Li N., Gügel I.L., Giavalisco P., Zeisler V., Schreiber L., Soll J., Philippar K. (2015). FAX1, a novel membrane protein mediating plastid fatty acid export. PLoS Biol..

[21] Schnurr J.A., Shockey J.M., De Boer G.-J., Browse J.A. (2002). Fatty acid export from the chloroplast. Molecular characterization of a major plastidial acyl-coenzyme a synthetase from arabidopsis. Plant Physiol..

[22] Xiao S., Chye M.L. (2011). New roles for acyl-CoA-binding proteins (ACBPs) in plant development, stress responses and lipid metabolism. Prog. Lipid Res..

[23] Chapman K.D., Ohlrogge J.B. (2012). Compartmentation of triacylglycerol accumulation in plants. J. Biol. Chem..

[24] Shockey J., Browse J. (2011). Genome-level and biochemical diversity of the acyl-activating enzyme superfamily in plants. Plant J..

[25] Groot P.H., Scholte H.R., Hülsmann W.C. (1976). Fatty acid activation: Specificity, localization, and function. Adv. Lipid Res..

[26] Shockey J.M., Fulda M.S., Browse J.A. (2002). Arabidopsis contains nine long-chain acyl-coenzyme a synthetase genes that participate in fatty acid and glycerolipid metabolism. Plant Physiol..

[27] Breuers F.K.H., Bräutigam A., Geimer S., Welzel U.Y., Stefano G., Renna L., Brandizzi F., Weber A.P.M. (2012). Dynamic remodeling of the plastid envelope membranes—A tool for chloroplast envelope in vivo localizations. Front. Plant Sci..

[28] Andrews J., Keegstra K. (1983). Acyl-CoA synthetase is located in the outer membrane and Acyl-CoA thioesterase in the inner membrane of pea chloroplast envelopes. Plant Physiol..

[29] Pongdontri P., Hills M. (2001). Characterization of a novel plant acyl-coA synthetase that is expressed in lipogenic tissues of Brassica napus L.. Plant Mol. Biol..

[30] Aznar-Moreno J.A., Venegas Calerón M., Martínez-Force E., Garcés R., Mullen R., Gidda S.K., Salas J.J. (2014). Sunflower (*Helianthus annuus* ) long-chain acyl-coenzyme A synthetases expressed at high levels in developing seeds. Physiol. Plant..

[31] Fulda M., Shockey J., Werber M., Wolter F.P., Heinz E. (2002). Two long-chain acyl-CoA synthetases from *Arabidopsis thaliana* involved in peroxisomal fatty acid β-oxidation. Plant J..

[32] Jessen D., Olbrich A., Knüfer J., Krüger A., Hoppert M., Polle A., Fulda M. (2011). Combined activity of LACS1 and LACS4 is required for proper pollen coat formation in Arabidopsis. Plant J..

[33] Weng H., Molina I., Shockey J., Browse J. (2010). Organ fusion and defective cuticle function in a lacs1 lacs2 double mutant of Arabidopsis. Planta.

[34] Schnurr J., Shockey J., Browse J. (2004). The Acyl-CoA synthetase encoded by LACS2 is essential for normal cuticle development in arabidopsis. Plant Cell.

[35] Lü S., Song T., Kosma D.K., Parsons E.P., Rowland O., Jenks M.A. (2009). Arabidopsis *CER8* encodes LONG-CHAIN ACYL-COA SYNTHETASE 1 (LACS1) that has overlapping functions with LACS2 in plant wax and cutin synthesis. Plant J..

[36] Fulda M., Schnurr J., Abbadi A., Heinz E., Browse J. (2004). Peroxisomal Acyl-CoA Synthetase Activity Is Essential for Seedling Development in Arabidopsis thaliana. Plant Cell.

[37] Zhao L., Katavic V., Li F., Haughn G.W., Kunst L. (2010). Insertional mutant analysis reveals that long-chain acyl-CoA synthetase 1 (LACS1), but not LACS8, functionally overlaps with LACS9 in Arabidopsis seed oil biosynthesis. Plant J..

[38] Ritzenthaler C., Nebenführ A., Movafeghi A., Stussi-Garaud C., Behnia L., Pimpl P., Staehelin L.A., Robinson D.G. (2002). Reevaluation of the effects of brefeldin a on plant cells using tobacco bright yellow 2 cells expressing golgi-targeted green fluorescent protein and copi antisera. Plant Cell.

[39] Mitsui T., Ochiai A., Yamakawa H., Kaneko K., Kitajima-Koga A., Baslam M. (2018). Novel molecular and cell biological insights into function of rice α-amylase. Amylase.

[40] Radif Y., Ndiaye H., Kalantzi V., Jacobs R., Hall A., Minogue S., Waugh M.G. (2018). The endogenous subcellular localisations of the long chain fatty acid-activating enzymes ACSL3 and ACSL4 in sarcoma and breast cancer cells. Mol. Cell. Biochem..

[41] Hiei Y., Ohta S., Komari T., Kumashiro T. (1994). Efficient transformation of rice (Oryza sativa L.) mediated by Agrobacterium and sequence analysis of the boundaries of the T-DNA. Plant J..

[42] Fukuoka H., Ogawa T., Mitsuhara I., Iwai T., Isuzugawa K., Nishizawa Y., Gotoh Y., Nishizawa Y., Tagiri A., Ugaki M. (2000). Agrobacterium-mediated transformation of monocot and dicot plants using the NCR promoter derived from soybean chlorotic mottle virus. Plant Cell Rep..

[43] Mitsui T., Yamaguchi J., Akazawa T. (1996). Physicochemical and serological characterization of rice α-amylase isoforms and identification of their corresponding genes. Plant Physiol..

[44] Kaneko K., Takamatsu T., Inomata T., Oikawa K., Itoh K., Hirose K., Amano M., Nishimura S.-I., Toyooka K., Matsuoka K. (2016). *N*-Glycomic and microscopic subcellular localization analyses of NPP1, 2 and 6 strongly indicate that *trans*-Golgi compartments participate in the Golgi to plastid traffic of nucleotide pyrophosphatase/phosphodiesterases in rice. Plant Cell Physiol..

[45] Takamatsu T., Baslam M., Inomata T., Oikawa K., Itoh K., Ohnishi T., Kinoshita T., Mitsui T. (2018). Optimized method of extracting rice chloroplast DNA for high-quality plastome resequencing and *de novo* assembly. Front. Plant Sci..

[46] Inomata T., Baslam M., Masui T., Koshu T., Takamatsu T., Kaneko K., Pozueta-Romero J., Mitsui T. (2018). Proteomics analysis reveals non-controlled activation of photosynthesis and protein synthesis in a rice *npp1* mutant under high temperature and elevated CO_2_ conditions. Int. J. Mol. Sci..

