# Supplementary material for: Functional Analysis of Rice Long-Chain Acyl-CoA Synthetase 9 (OsLACS9) in the Chloroplast Envelope Membrane
Source: Int J Mol Sci. 2020 Mar 23;21(6):2223. doi: 10.3390/ijms21062223 (PMC7139535; doi:10.3390/ijms21062223)
Supplement: Supplementary file 1 [file ijms-21-02223-s001.zip › v3R2 LACS9 SupFigTab/R2_Spplemental Figures1-4_LACS9_Round2 - .pdf]

Supplemental Figure 1

A

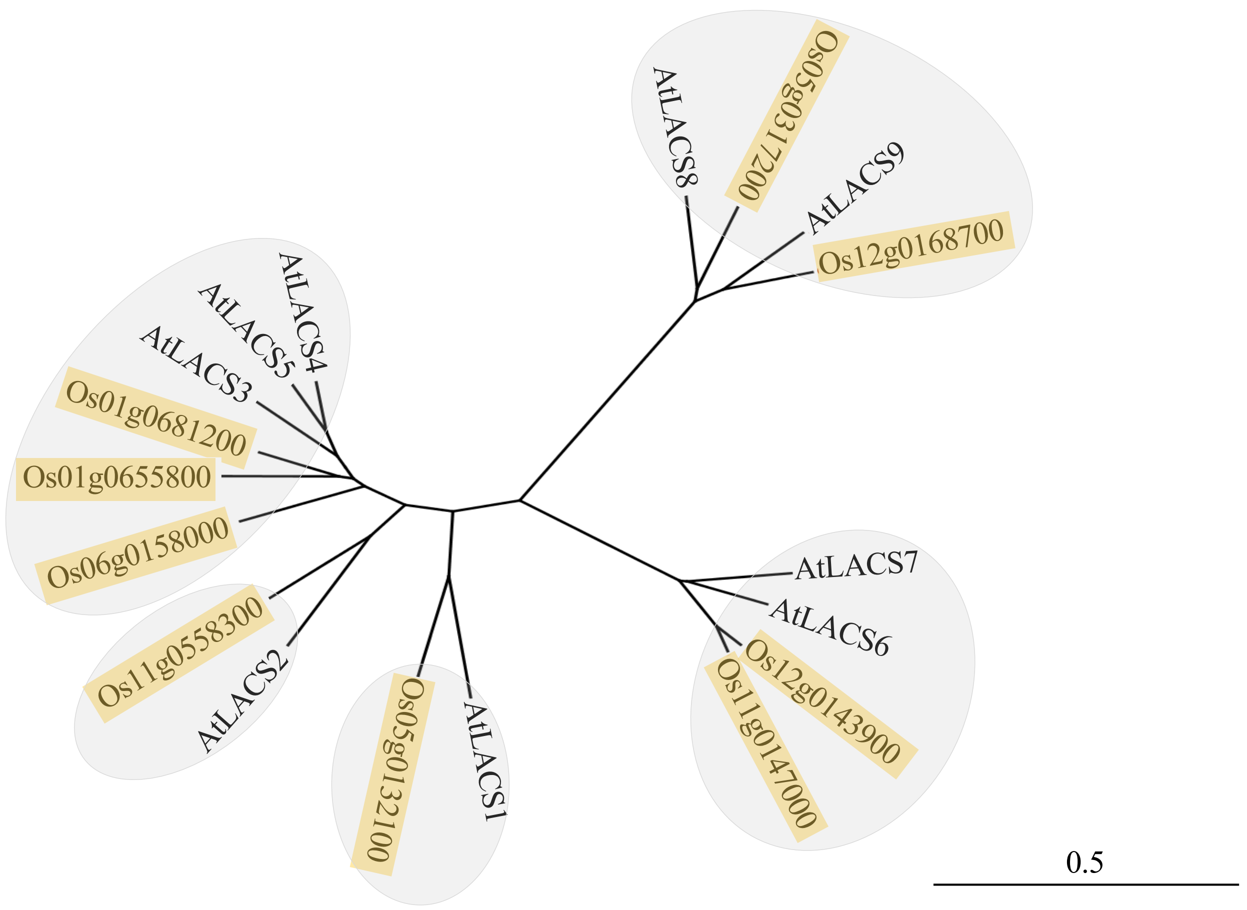

B

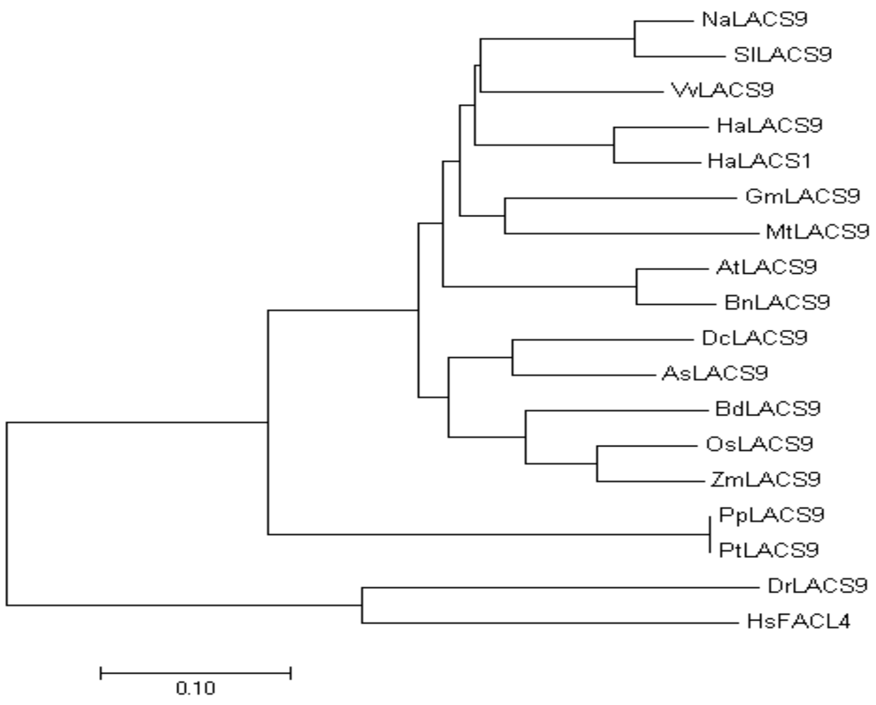

Supplemental Figure 1

Amino acid sequence analysis of OsLACS9 proteins

(A) Phylogenetic tree comparison of *Oryza sativa* (yellow box) and *Arabidopsis LACSs* genes. CLC sequence viewer v. 8.0 software (https://www.qiagenbioinformatics.com) was used for construction of phylogenetic trees by neighbor-joining methods. The scale bar represents 0.5 substitutions per amino acid residue.

(B) Phylogenetic comparison of LACS9 proteins from different species. The phylogenetic tree of the multiple sequence alignment of LACS proteins was constructed by neighbor-joining algorithms using ClustalW in MEGA 7 under the default setting. The phylogenetic tree includes the following plant species: **As**, *Apostasia shenzhenica* (Protein ID: A0A2I0B156); **At**, *Arabidopsis thaliana* (Protein ID: Q9CAP8); Bd, *Brachypodium distachyon* (Protein ID: I1IN37); **Bn**, *Brassica napus* (Protein ID: NM_001316278); **Dc**, *Dendrobium catenatum*; **Dr**, *Danio rerio* (Protein ID: NP_001139404.1); **Gm**, *Glycine max* (Protein ID: XM_003542913); **Ha**, *Helianthus annuus*; **Hs**, *Homo sapiens* (Protein ID: Y12777); **Mn**, *Medicago truncatula* (Protein ID: XM_003601977); **Na**, *Nicotiana attenuate* (Protein ID: A0A314L741); **Os**, *Oryza sativa* (Protein ID: NP_177882.1); **Pp**, *Physcomitrella patens* (Protein ID: A9T939); **Pt**, *Populus trichocarpa* (Protein ID: A9T939); **Sl**, *Solanum lycopersicum* (Protein ID: XM_004250346); **Vv**, *Vitis vinifera* (Protein ID: E0CR69); **Zm**, *Zea mays* (Protein ID: XM_008664290). The zebrafish (*Danio rerio*) and human (*Homo sapiens*) sequences were selected from homologous Vertebrate sequences and used as an outgroup to root the tree.

Supplemental Figure 2

```
1    MNPYFVGLLVPIAVSLLLRKRRMVGKMRALPVDVGGEPGYAIRNYRFKQP
51   VETHWEGVTTLAELFEQSCKDYVNMPLLGTRKLISREQESSLDGRSFEKL
101  HLGEYDWKCYAEVFKSVCNFASGLIRLGHQKTDRVAIFAETRAEWQIALQ
151  ACFRQNITVVTIYASLGEEALCHSLNETEVTTVVCGQKELKKMIDISGQL
201  DTVKRVIYINEEGISAEVSLAQKSTSWIIEPFEDVGRLGDTAPVDANMPL
251  PSDVAVIMYTSGSTGLPKGVMMTHRNVLATLSAVMTIVPEIGKKDVYLAY
301  LPLAHILELAAEALIAAVGASIGYGSPLTLTDTSNKIKKGTLGDASALKP
351  TLMTAVPAILDRVRDGVRKKVDTKGGVAKQLFDVAYNRRLAAVNGSWLGA
401  WGLEKLLWDMLVFKKVRAVLGGKIRFVLSGGAPLSGDTQRFINICLGVPI
451  GQGYGLTETCAGGTFSEYDDPSVGRVGAPLPCSYIKLIDWSEGGYLTSDS
501  PMPRGEIVIGGPNVTKGYFKNEAKTNEVYKDDEKGMRWFYSGDIGRFHPD
551  GCLEIIDRKKDIVKLQHGEYVSLGKVEAALIVSPYVENIMIHADPFHSYC
601  VALVVAAHNELENWASQQGVTYTDFVDLCQKPEAVKEVLGSLSKAAKQAR
651  LEKFEIPAKIKLISEPWTPESGLVTAALKLKREVLRKTYEDDLAKLYA
```

Structural organization of OsLACS9.

Blue box shows predicted N-terminal ER signal sequence, orange shows transmembrane domains, and green box shows *N*-glucosylation sites.

Supplemental Figure 3

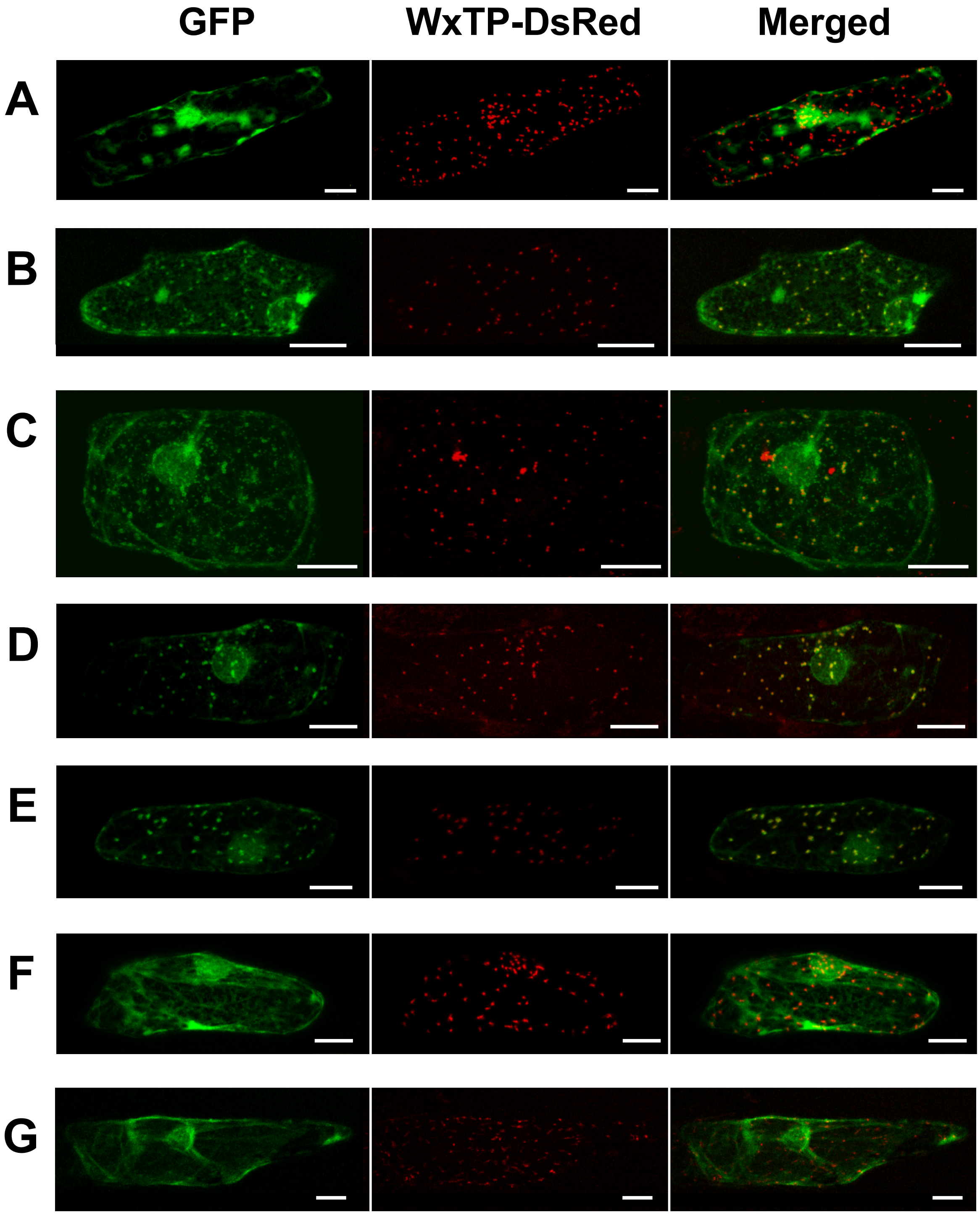

Localization of each truncated OsLACS9-GFP and WxTP-DsRed (plastid stroma marker) fusion protein co-bombarded in onion epidermal cells.

A, GFP alone; **B,** OsLACS9(full)-GFP; **C,** OsLACS9(300a.a.)-GFP; **D**, OsLACS9(100a.a.)-GFP; **E**, OsLACS9(70a.a.)-GFP; **F**, OsLACS9(20a.a.)-GFP; **G**, OsLACS9(ΔSP)-GFP.

Bars = 50 µm.

Supplemental Figure 4

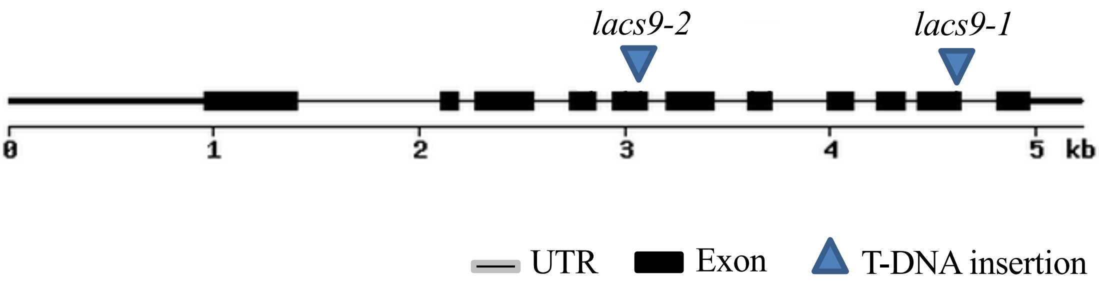

Genetic information on rice *lacs9-1* and *lacs9-2*.

Gene-structure of *LACS9* highlighting location of the T-DNA insertion site within *lacs9-1* (NE3550) and *lacs9-2* (NF6026) mutants.
